# Therapeutic hypothermia can cause non-infective C-reactive protein elevating

**DOI:** 10.3389/fped.2023.1157417

**Published:** 2023-04-24

**Authors:** Xiaohong Wang, Liping Shi, Chenhong Wang, Xiaolu Ma

**Affiliations:** Department of Neonatology, Children’s Hospital, Zhejiang University School of Medicine, National Clinical Research Center for Child Health, Hangzhou, China

**Keywords:** therapeutic hypothermia(TH), hypoxic-ischemic encephalopathy (HIE), c-Reactive protein, brian injury, neonatal

## Abstract

**Objective:**

To analyze the relationship between therapeutic hypothermia (TH) and whole blood high-sensitivity C-reactive protein (hs-CRP) in neonates with hypoxic-ischemic encephalopathy (HIE).

**Method:**

Retrospective analysis was made on the clinical data of hospitalized infants diagnosed with asphyxia in our neonatal intensive care unit from January 2014 to June 2021. According to whether TH was performed, they were divided into two groups, the control group (missed the time in other hospitals and did not receive TH) and the treatment group (TH group). In their first ten days, analysis was made on the hs-CRP, white blood cell (WBC) count, neutrophil percentage, platelet count (PLT), and brain MRI. The correlation analysis was carried out based on the severity of brain injury displayed by the brain MRI and the time of hs-CRP elevation to summarize the relationship between TH and the time of hs-CRP elevation and the severity of HIE.

**Results:**

83 infants were included, 28 in the control group and 55 in the TH group. After birth, 33 infants (60.0%) in the TH group and 2 patients (7.1%) in the control group had elevated hs-CRP, which was statistically significant (*P* < 0.05). The time window for CRP elevation after TH was 72–96 h after the end of treatment; The results of the brain MRI showed 23 in the TH group and 11in the control group with moderate and severe HIE. 21 infants (all in the TH group) had elevated hs-CRP. MRI showed that the number of infants with mild injury or regular infants whose hs-CRP raised in the TH group was 12, and the rate of hs-CRP elevation was 37.5%; in the control group, the rate was 11.8%. The difference was significant. TH can decrease PLT and WBC, but no significance in the two groups. Blood and sputum cultures were negative in all infants, and there were no signs of infection.

**Conclusions:**

TH can increase the blood hs-CRP of HIE neonates, and the probability of its occurrence is related to the severity of HIE. The heavier the HIE, the higher the risk of hs-CRP elevation after TH; The hs-CRP elevation has little to do with infection, and it doesn't recommend using antibiotics actively.

## 1. Introduction

C-reactive protein (CRP) is an acute-phase protein produced predominantly by hepatocytes. Serum CRP is elevated in response to acute infection, inflammatory conditions and trauma and increases with age (1). In clinical practice, standard and high-sensitivity CRP (hs-CRP) assays can determine serum CRP levels. However, the standard assays are insufficiently sensitive to measure the low grade CRP, especially in newborns, because their liver and immune system are underdevelopment. By contrast, measurement of hs-CRP levels can accurately detect low-grade inflammatory states.

Hypoxic-ischaemic encephalopathy (HIE) is a type of neonatal encephalopathy caused by systemic hypoxemia and/or reduced cerebral blood flow resulting from an acute peripartum or intrapartum event (2). It can be a clinical consequence of perinatal, birth and/or neonatal asphyxia. In developed countries, the incidence rate of moderate to severe neonatal HIE is about 1 ‰ to 3 ‰, of which about 15% of the children die in the neonatal period (3, 4). About 30% of the surviving children left long-term complications and sequelae of different types and degrees, such as cognitive impairment, motor development backwardness, and even cerebral palsy (5). Clinical studies in the past decades have shown that therapeutic hypothermia (TH: target temperature 33–34°C) significantly protects hypoxic-ischemic brain nerves and organs and can enormously improve the survival rate and disability rate of full-term neonates with moderate to severe HIE (6). The follow-up found that the prognosis of HIE patients who were treated with TH was significantly improved compared to untreated asphyxia children (7). Presently, TH of neonatal HIE has been widely used in our country and abroad. However, TH often produces adverse effects, such as abnormal coagulation function, hypotension, pulmonary hypertension, thrombocytopenia, hyperlactatemia, arrhythmia, infection, etc. (8). These may even lead to the death of the treated children in severe cases. Due to the more common and apparent complications, such as blood hyperviscosity syndrome, thrombocytopenia, and hyperlactatemia caused by TH in children, they are generally treated promptly and effectively. However, there are few studies on the hs-CRP level of infants caused by TH. Many hospitals and doctors give antibiotics to children after their blood hs-CRP rises, which is inappropriate (9). So we need to effectively judge the risk of infection in children treated with mild hypothermia and take reasonable measures according to the evaluation results for the hs-CRP increase in the TH. On the one hand, it can avoid unreasonable use of antibiotics and reduce the adverse side effects of children's use of antibiotics; on the other hand, it can shorten the hospital stay and reduce the burden on families and society. Given the above reasons, we analyzed the clinical data of children hospitalized in our NICU from January 2014 to June 2021 diagnosed with neonatal asphyxia and eligible for mild hypothermia treatment to find the relationship between TH of neonatal HIE and whole blood hs-CRP.

## 2. Method

### 2.1. Data method

**Figure 1:**
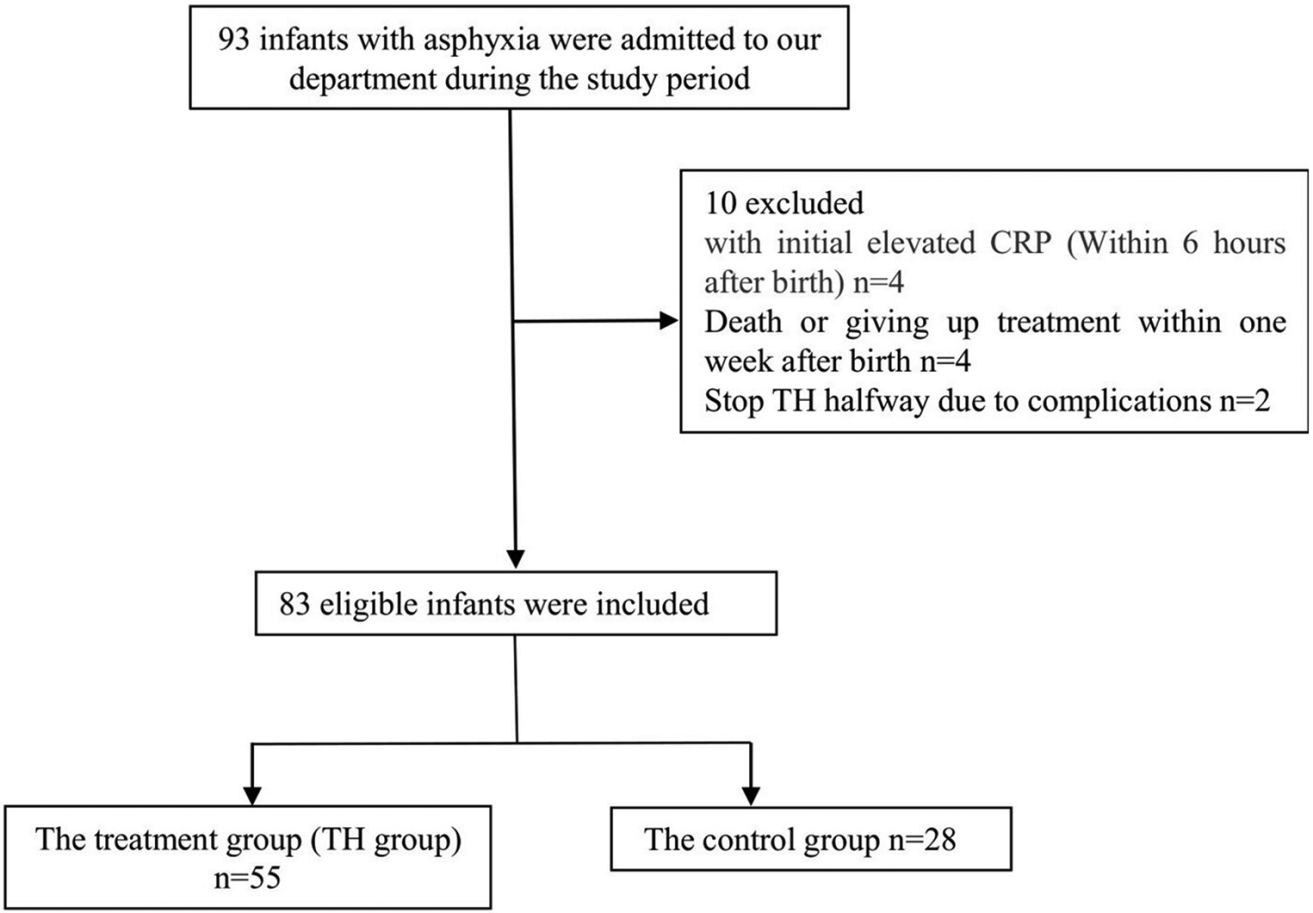
Flowchart of included patients.

Infants diagnosed with neonatal asphyxia in our NICU department from January 2014 to June 2021 and eligible for TH were selected (including those who were suitable for TH for postnatal asphyxia but did not receive treatment locally and missed the treatment time in our hospital). The TH criteria are as follows: the baby is ≥35 weeks, and ≥1,800 grams, and less than 6 h old and presents with evidence of acute perinatal/intrapartum hypoxia-ischemia as suggested by at least one of the following: (A) Apgar score ≤5 at 10 min, (B) Blood gas (cord/arterial/venous/capillary) within 60 min of birth includes either a: pH < 7.0, or Base excess equal to or worse than minus 12 mmol/L, (C) Ongoing resuscitation for ≥10 min (10). Exclusion criteria: infant with high-risk factors of perinatal infection (such as premature rupture of membranes more than 18 h, infection of the mother, mother having a fever or other signs of infection), congenital malformation, and death within three days after birth. A total of 83 eligible infants were included, including 42 girls and 41 boys. The selected infants were divided into the control and TH groups according to whether they had TH. There were 55 patients in the TH group (rectal temperature 33°C–34°C) and 28 in the control group (Figure 1). We used the online epidemiological calculation website established by Ausvet Animal Health Service, funded by the Australian Cooperative Research Center for Biosafety, to verify our sample size, the power (1−*β*) is greater than 0.8, so we think our sample size is appropriate (https://epitools.ausvet.com.au/). The TH group infants were cooled using a commercially made device (Blanketrol II. Cincinnati Sub-Zero Inc. OH, USA.), whole-body cooling was chosen to induce TH, with core temperature (rectal) maintained around 33–34°C for 72 h. After 72 h of cooling, rewarm the baby at a rate not exceeding 0.5°C every 2 h, and the target rectal temperature is 37°C. Collect the primary data of the two groups, including the factors that may affect hs-CRP, such as patient's sex, birth weight, birth gestational age, Apgar score, amniotic fluid(AF), ventilator use or not, blood leukocyte count, neutrophil percentage, platelet count, duration of antibiotic use, the hs-CRP value within ten days after birth, and brain Cranial magnetic resonance imaging(MRI) data within 4–8 days after delivery. We chose 3 days as the cut-off of the ventilator based on a previous study (11). In the control group, the blood data were collected from their birth hospital in the first or second days. According to the literature, the hs-CRP >10 mg/L is considered to be increased (12). Since the lower limit of the laboratory monitoring of the hs-CRP is <0.5 mg/L, <0.5 mg/L wa calculated as 0.5 mg/L in statistics and mapping analysis. The BC-7500 Auto Hematology and CRP analyzer and supporting calibrators (Shenzhen Mindray Corporation, Shenzhen, China.) were used to measure CBC and hs-CRP. We used the latex particle-enhanced immunonephelometry method to measure hs-CRP. EDTA-K2 anti-coagulated whole blood samples were collected from both groups. High sensitivity C-Reaction Protein (hs-CRP) reagent mixed with the lysed sample, and the antibody-marked latex microcell in the latex reagent undergoes agglutination reaction with the hs-CRP. The reaction will increase the solution's turbidity. The turbidity of the solution increased, and thus the hs-CRP concentration was gained by the turbidity analysis (13). MRI was classified as mild, moderate, and severe according to the Barkovich score (14).

1.2 According to a previous report, infection was defined as meeting one of the following criteria: (1) septicemia, (2) septic shock, or (3) administration of antibiotics for ≥10 days (15).

### 2.2. Statistical methods

IBM SPSS Statistics 23 statistical software was used for data analysis. *T*-test was used to compare the mean between the two groups, the *χ*^2^ test was used to compare the rates between the two groups, and logistic regression analysis was used to analyze the risk factors related to hs-CRP increase, inspection level *α* = 0.05.

## 3. Result

There were 83 patients in the two groups, including 28 in the control group and 55 in the treatment group. The general data of the patient's sex, gestational age, birth weight, Apgar score, delivering pattern, premature rupture of membranes, etc., were shown in Table 1. There was no significant difference in sex ratio, gestational age at birth, birth weight,birth Apgar score, delivering pattern, and premature rupture of membranes between the two groups. All enrolled children's sputum and blood cultures were negative, with no fever or other abnormalities. The *χ*^2^ test showed no significant difference in the II–III °AF pollution at birth between the control and TH groups, which was 35.7% and 40%, respectively, *Χ*^2^ = 0.144, *P* = 0.7 (Table 2A). There was also no significant difference between the two groups in the proportion of patients who had used a ventilator for more than three days, 39.3% and 38.2%, respectively, *Χ*^2^ = 0.01, *P* = 0.92 (Table 2B). The total number of patients with elevated hs-CRP in the two groups was 35, including 2 in the control group; the soaring rate of hs-CRP was 60.0%. In the treatment group, the number of patients with elevated hs-CRP was 33, and the high rate of CRP was 60.0%, which was statistically significant, *Χ*^2^ = 21.56, *P < *0.001 (Table 3A). The results of head MR showed that there were 34 patients with moderate and severe HIE, of which 21 patients (all children in the treatment group) had elevated hs-CRP, and the incidence of moderate and severe HIE elevated hs-CRP was 61.76%. In the treatment group, the increased rate of hs-CRP in children with moderate to severe HIE and normal or mild HIE was 91.3% and 37.5%, respectively, with a statistical difference, *Χ*^2^ = 16.14, *P < *0.001 (Table 3B). In the control group, there was no statistical difference in the increased rate of hs-CRP between children with moderate to severe HIE and those with normal or mild HIE. Fisher's exact test showed that *P* = 0.51 (see Table 3C). The multivariate logistic regression equation was constructed by amniotic fluid, head MR, mild hypothermia treatment, and duration of ventilator use. The results showed that the influence of amniotic fluid turbidity on hs-CRP was statistically significant (OR = 3.89, 95% CI 1.11–13.65, *P* = 0.034). The risk of hs-CRP increase in children with amniotic fluid II–III° turbidity was nearly 2.89 times higher than those with amniotic fluid clear or I° turbidity; The severity of head MR in HIE children had a significant impact on hs-CRP (OR = 5.0, 95% CI: 1.39–17.9, *P* = 0.014). The risk of hs-CRP elevation in children with moderate to severe head MR abnormalities was four times higher than in children with normal or slightly abnormal head MRI. TH significantly impacted hs-CRP (OR = 40.54, 95% CI: 6.15–267.39, *P* < 0.001). The risk of hs-CRP increase in children with mild hypothermia treatment was nearly 39.54 times higher than in children without treatment. TH was the leading risk factor for hs-CRP growth. There was no significant difference in hs-CRP elevation between children who did not use a respirator or used a respirator ≤3 days and those who used it more than three days (OR = 3.24 95% CI: 0.87–12.07, *P* = 0.08) (Table 4).

**Table 1:** Baseline characteristics and clinical data of patients for entire study group.

Variable	Control (*n* = 28)	Treatment (*n* = 55)	*P* Value
Sex (man/woman)	15/13	26/29	−
Gestation weeks(mean ± SD)	38.9 ± 1.73	39.2 ± 1.39	0.447
Birth weight g(mean ± SD)	3040.1 ± 579.02	3172 ± 454.24	0.2709
Delivering pattern (*n*) (spontaneous delivery/cesarean section)	8/20	27/28	0.12
Premature rupture of membranes (*n*)	6	12	0.60
Ventilator ≤3 days/> 3 days (*n*)*	17/11	34/21	0.92
Amniotic fluid clear-I°/II–III° (*n*)	18/10	33/22	0.7
Apgar score(median)			
1 min	3 (0–6)	2 (0–8)	0.4657
5 min	5 (0–9)	5 (0–9)	0.9757
10 min	7 (1–10)	6 (1–9)	0.2690

* We chose 3 days as the cut-off of the ventilator based on a previous study (11).

**Table 2:** Comparison of high-risk factors between the two groups.

(A). Comparison of amniotic fluid between the two groups					
Variable	Total *n*	AF clear-I° *n* (%)	AF 2–III° *n* (%)	*Χ* ^2^	*P*
Control	28	18 (64.3)	10 (35.7)	0.144	0.70
Treatment	55	33 (60)	22 (40)		
(B). Comparison of the duration of ventilator use between the two groups					
Variable	Total *n*	NO/Ventilator ≤3D *n* (%)	Ventilator > 3D *n* (%)	*Χ* ^2^	*P*
Control	28	17 (60.7)	11 (39.3)	0.01	0.92
Treatment	55	34 (61.8)	21 (38.2)		

**Table 3:** Comparison of hs-CPR in different and within the groups.

(A). Comparison of hs-CRP elevation rate between the two groups					
Variable	Total *n*	CRP elevated *n* (%)	CRP Normal *n* (%)	*Χ* ^2^	*P*
Control	28	2 (7.1)	26 (92.9)	21.56	.000
Treatment	55	33 (60.0%)	22 (40.0%)		
(B). Comparison of hs-CRP elevation rate with different degrees of brain injury in the TH group					
Brain Injury	Total *n*	CRP elevated *n* (%)	CRP Normal *n* (%)	*Χ* ^2^	*P*
Moderate or severe	23	21 (91.3)	2 (8.7)	16.14	.000
Normal or mild	32	12 (37.5)	20 (62.5)		
(C). Comparison of hs-CRP elevation rate with different degrees of brain injury in the Control group					
Brain Injury	Total *n*	CRP elevated *n* (%)	CRP Normal *n* (%)	*P*	
Moderate or severe	11	0 (0.0)	11 (100)	0.51	
Normal or mild	17	2 (11.8)	15(88.2)		

**Table 4:** Multivariate logistic regression analysis results*.

Variable	B	S.E.	Wald	*P*	OR	OR (95%CI)
Intercept	−4.76	1.16	16.72	.000	0.01	
AF	1.36	0.64	4.52	0.034	3.89	1.11–13.65
Brain MRI	1.61	0.65	6.08	0.014	4.99	1.39–17.90
TH	3.70	0.96	14.80	.000	40.54	6.15–267.39
Ventilation	1.18	0.67	3.07	0.08	3.24	0.87–12.07

* CI, confidence interval; OR, odds ratio.

**Figure 2:**
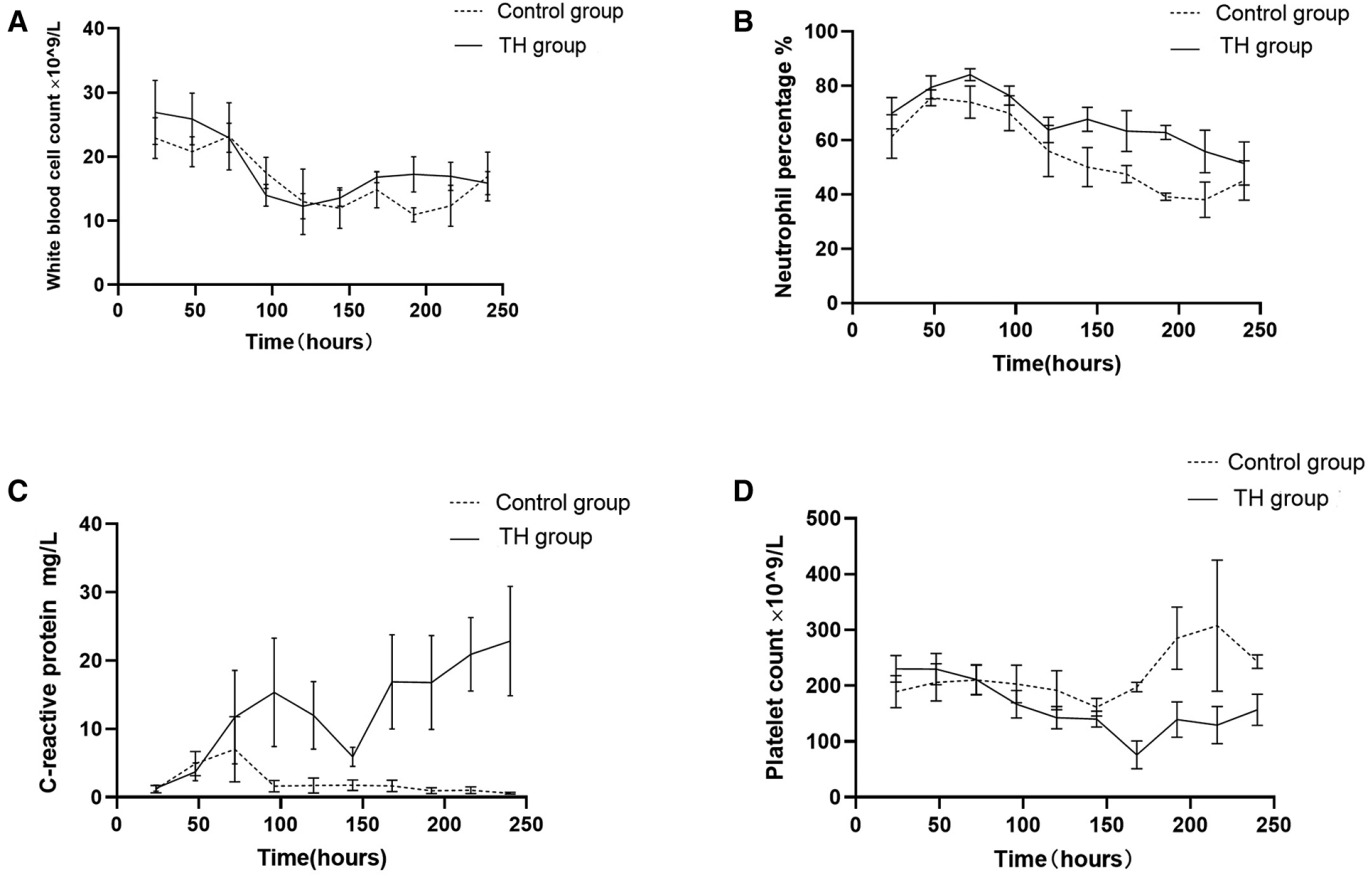
Changes of blood routine indexes and hs-CPR in children from birth to 10 days in life. (**A**) The white blood cell count decreased slowly with the increase of age and tended to be stable after 96 h of birth in both groups, with no significant difference between them. (**B**) The percentage of neutrophils changed similarly in both groups, but it was slightly higher in the treatment group within the observation time (10 days). (**C**) The hs-CRP rises sharply within 48 h after birth in the treatment group. It first peaked at 72–96 h. On the 6th postnatal day (144 h), the hs-CRP began to decline. (**D**) Platelet in the treatment group is lower than that in the control group after TH.

**Figure 3:**
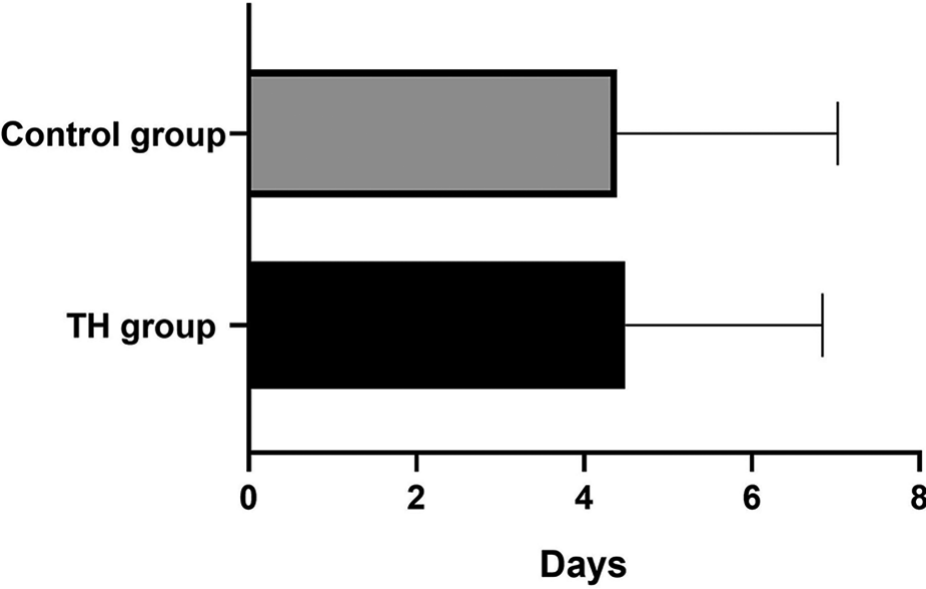
The use of antibiotics in the two groups. The duration was (4.49 ± 2.35) and (4.39 ± 2.63) days, respectively, *P > *0.05.

The white blood cell count (WBC) of all children decreased slowly with the increase of age and tended to be stable after 96 h of birth. There was no significant difference in the WBC between the two groups (Figure 2A). The percentage of neutrophils in the THgroup was higher than the control group within the observation time (10 days), *t* = 5.75, *P* < 0.001, 95% CI: 7.135–16.39 (Figure 2B). Compared to control group, the hs-CRP value of 60% of children in the TH group began to rise within 48 h after birth. It first peaked at 72–96 h (18.83 ± 3.86 mg/L). On the 6th postnatal day (144 h), the hs-CRP began to decline. It remained slightly higher than 12.5 ± 2.05 mg/L, with a significant difference between the two groups (*P* < 0.05) (Figure 2C). The increase of hs-CRP caused by mild hypothermia can last for 10 days, and some can last for almost 2 weeks before falling to normal. The platelet levels in the THgroup and the control group within 72 h after birth were (208.47 ± 19.56) × 10^9^/L and (224.35 ± 14.36) × 10^9^/L, respectively. The two groups had no significant difference, *t* = −0.665, *P* = 0.511, 95% CI (−64.67 to 32.90). After 72 h, PLT in the treatment group was generally lower than in the control group, with a statistical significance of *P* < 0.05 (Figure 2D). Blood and sputum cultures were negative in all infants, and there were no signs of infection in all infants.Due to high-risk factors such as premature rupture of membranes and turbid amniotic fluid in some infants, penicillin or third-generation cephalosporins were empirically used upon admission. The number of patients in the TH group using antibiotics was 48, while the number in the control group was 23. The days of antibiotic use were (4.49 ± 2.35) and (4.39 ± 2.63), respectively, no significant difference, see Figure 3.

## 4. Discussion

HIE is a severe disease of neonatal that occurs in 2.5 per 1,000 live term births. It can be a clinical consequence of perinatal and birth neonatal asphyxia (3). It is a condition which can cause significant mortality and long-term morbidity, negatively impacting the patient's quality of life and resulting in a substantial financial burden (16). Currently, the therapies for these patients include good supportive care such as assisted ventilation, cardiovascular support, and erythropoietin application (17). But TH is the only proven treatment for moderate and severe HIE that improves mortality and long-term outcomes for babies at present (18).

However, TH has many adverse effects, such as hypotension, apnoea, thrombocytopenia, and infections (19). In previous studies, immunosuppression has been demonstrated in babies receiving TH (20). Elevated CRP is considered as an essential indicator of infection, including bacterial and viral infection and chronic inflammation. In recent years, the role of CRP as a medium for tissue damage has received increasing attention, there is strong evidence for this in some animal species and human being for a long time (21–23). In the present study, we analyzed the relationship between the elevated hs-CRP and infection in HIE infants. We found a significant increase in hs-CRP and a decrease in platelet in HIE infants treated with TH. The rise in hs-CRP in the first four days of life is consistent with a previous report (24), But our research shows that hs-CRP drops once on day sixth; it is still higher than the control group at the same time point, we think this decrease is likely related to sample size. No other signs of infection were found. Those with elevated hs-CRP infants did not meet the criteria of infection; no one used antibiotics for more than ten days or had a shock. As we all know, CRP is synthesized in hepatocytes (25), and it is partly regulated by a self-mediated protease released from neutrophils promoting auto-gradation. Its main immunological functions are the opsonization of biological particles (bacteria and dead or dying cells) for their clearance by macrophages and the activation of the classical complement pathway. This helps to eliminate pathogens and dead cells (26, 27). In clinical work, we are used to linking the increase of CRP with inflammation, which can result in prolonged antibiotic exposure. But in recent years, our understanding of what can elicit inflammation has expanded considerably. We have learned that the innate immune system responds to metabolic stress with chronic low-grade inflammation (“metaflammation”) without the classic signs of acute inflammation (28). One molecular mechanism that can trigger low-grade inflammation and CRP induction in response to metabolic stress that has been well-studied is the unfolded protein response (29). Barrett TD, et al. found that CRP is associated with an increase in myocardial infarct size after ischemia/reperfusion (30). Mild hypothermia can lead to a rise in blood lactic acid, which has a specific toxic effect on myocardial cells and may increase CRP. In addition, hypothermia's inhibitory effect on enzymes such as proteases might account for CRP's longevity, which may also cause CRP to be higher than usual (31). Previous studies have shown that CRP can promote atherosclerosis, blood stagnation, and plaque formation. TH can lead to blood hyperviscosity syndrome, so the increase in CRP may be related to this reason (32, 33). According to the brain MR results and the elevated CRP in both groups in our research, we believe that the heavier the HIE, the higher the risk of hs-CRP elevation after mild hypothermia treatment. This phenomenon may be due to the more severe HIE, the more damaged cells, and the more powerful the stress response. Therefore, CRP is higher (34). Our research found that TH led to an increase in hs-CRP, but these newborns showed no other signs of infection. They had no fever, apnea, or positive etiological findings. This subclinical inflammation occurs in many conditions with minor degrees of metabolic dysfunction and stress state (28, 29, 35). There is no evidence that antibiotics must be used in this kind of baby. Platelet count was lower in severely asphyxiated children. TH can lead to an increase in the proportion of neutrophils, which may be related to stress.

The limitation of this study may include the following aspects. Firstly, this is a retrospective study in nature, and the sample of the study is relatively small. Secondly, some variables were not evaluated in the study, such as IL-6 and TNF-α. Thus, a prospective study with large samples is warranted in the future. Nonetheless, to our knowledge, the present study is precious in confirming infection or not in HIE with TH. This information could help guide the use of antibiotics.

In conclusion, TH significantly affects the postnatal course of inflammatory markers, including hs-CRP response and lower platelet count. But there is not enough evidence that these changes indicate infection. Elevated hs-CRP can affect the evaluation of infection, leading to inappropriate antibiotic use or prolonged courses of antibiotics. Our study provides information to inform antibiotic stewardship practice in this population.

## Data Availability

The raw data supporting the conclusions of this article will be made available by the authors, without undue reservation.

## References

[1] Pepys MB Hirschfield GM. C-reactive protein: a critical update. J. Clin. Invest. (2003) 111(12):1805–12. 10.1172/JCI200318921 12813013 PMC161431

[2] Verklan M Walden M. Hypoxic-ischaemic encephalopathy. In: Verklan M Walden M, editors. Core curriculum for neonatal intensive care, 5th ed., Saint Louis: Elsevier (2015). p. 761–5.

[3] Gale C Statnikov Y Jawad S Uthaya SB Modi N. Neonatal brain injuries in England: population-based incidence derived from routinely recorded clinical data held in the national neonatal research database. Arch Dis Child Fetal Neonatal Ed. (2018) 103:F301–6. 10.1136/archdischild-2017-313707 29180541 PMC6047140

[4] Battin M Sadler L Masson V Farquha C. Neonatal encephalopathy in New Zealand: demographics and clinical outcome. J Paediatr Child Health. (2016) 52(6):632–6. 10.1111/jpc.13165 27148886

[5] Graham EM Ruis KA Hartman AL Northington FJ Fox HE. A systematic review of the role of intrapartum hypoxia-ischemia in the causation of neonatal encephalopathy. Am J Obstet Gynecol. (2008) 199(6):587–95. 10.1016/j.ajog.2008.06.094 19084096

[6] Tagin MA Woolcott CG Vincer MJ Whyte RK Stinson DA. Hypothermia for neonatal hypoxic ischemic encephalopathy: an updated systematic review and meta-analysis. Arch Pediatr Adolesc Med. (2012) 166(6):558–66. 10.1001/archpediatrics.2011.1772 22312166

[7] Jia W Lei X Dong W Li Q. Benefits of starting hypothermia treatment within 6 h vs. 6–12 h in newborns with moderate neonatal hypoxic-ischemic encephalopathy. BioMed Central Pediatrics. (2018) 18(1):50. 10.1186/s12888-018-1610-5 29433475 PMC5809807

[8] Lemyre B Chau V. Hypothermia for newborns with hypoxic-ischemic encephalopathy. Paediatr Child Health. (2018) 23(4):285–91. 10.1093/pch/pxy028 30657134 PMC6007306

[9] Macallister K Smith-Collins A. Serial C-reactive protein measurements in newborn infants without evidence of early-onset infection. Neonatology. (2019) 116(1):85–91. 10.1159/000497237 31112949

[10] Jacobs SE Berg M Hunt R Tarnow-Mordi WO Inder TE Davis PG. Cooling for newborns with hypoxic ischaemic encephalopathy. Cochrane Database Syst Rev. (2013) 2013(1):CD003311. 10.1002/14651858.CD003311.pub3 23440789 PMC7003568

[11] He Q Wang W Zhu SC Wang MQ Kang Y Zhang R The epidemiology and clinical outcomes of ventilator-associated events among 20,769 mechanically ventilated patients at intensive care units: an observational study. Crit Care. (2021) 25(1):44. 10.1186/s13054-021-03484-x 33531078 PMC7851639

[12] Benitz WE Han MY Madan A Ramachandra P. Serial serum C-reactive protein levels in the diagnosis of neonatal infection. Pediatrics. (1998) 102:E41. 10.1542/peds.102.4.e41 9755278

[13] Lin Z Lin Q Yu PL Chen ZX Lin HF Zhu B Performance evaluation of routine blood and C-reactive protein analysis using Mindray BC-7500 CRP auto hematology analyzer. Ann Transl Med. (2022) 10(10):588. 10.21037/atm-22-1642 35722426 PMC9201145

[14] Barkovich AJ Hajnal BL Vigneron D Sola A Partridge JC Allen F Prediction of neuromotor outcome in perinatal asphyxia: evaluation of MR scoring systems. AJNR Am J Neuroradiol. (1998) 19(1):143–9. PMID: ; PMCID: PMC8337350 9432172 PMC8337350

[15] Smilga A-S Garfinkle J Ng P Andersen J Buckley D Fehlings D Neonatal infection in children with cerebral palsy: a registry-based cohort study. Pediatr Neurol. (2018) 80:77–83. 10.1016/j.pediatrneurol.2017.11.006 29428154

[16] Bryce J Boschi-Pinto C Shibuya K Black RE. WHO Child health epidemiology reference group, WHO estimates of the causes of death in children. Lancet. (2005) 365:1147–52. 10.1016/S0140-6736(05)71877-8 15794969

[17] Wu YW. Clinical features, diagnosis, and treatment of neonatal encephalopathy. Waltham, MA: UpToDate Inc (2020). Available at: https://www.uptodate.com (Cited September 28, 2020).

[18] Greenwood A Evans J Smit E. New brain protection strategies for infants with hypoxic-ischaemic encephalopathy. Paediatr Child Health (Oxford). (2018) 28(9):405–11. 10.1016/j.paed.2018.06.004

[19] Queensland Clinical Guidelines. Hypoxic ischaemic encephalopathy (HIE). Guideline No. MN21.11-V10-R26. Queensland Health (2021). Available at: http://www.health.qld.gov.au/qcg

[20] Jenkins DD Lee T Chiuzan C Perkel JK Rollins LG Wagner CL Altered circulating leukocytes and their chemokines in a clinical trial of therapeutic hypothermia for neonatal hypoxic-ischemic encephalopathy. Pediatr Crit Care Med. (2013) 14(8):786–95. 10.1097/PCC.0b013e3182975cc9 23897243

[21] Sheriff A Schindler R Vogt B Abdel-Aty H Unger JK Bock C Selective apheresis of C-reactive protein: a new therapeutic option in myocardial infarction? J Clin Apher. (2015) 30(1):15–21. 10.1002/jca.21344 25044559

[22] Szalai AJ Briles DE Volanakis JE. Role of complement in C-reactive-protein mediated protection of mice from streptococcus pneumoniae. Infect Immun. (1996) 64:4850–3. 10.1128/iai.64.11.4850-4853.1996 8890251 PMC174457

[23] Saito J Shibasaki J Shimokaze T Kishigami M Ohyama M Hoshino R Temporal relationship between Serum levels of interleukin-6 and C-reactive protein in therapeutic hypothermia for neonatal hypoxic-ischemic encephalopathy. Am J Perinatol. (2016) 33(14):1401–6. 10.1055/s-0036-1583192 27167641

[24] Perrone S Szabó M Bellieni CV Longini M Bangó M Kelen D Whole body hypothermia and oxidative stress in babies with hypoxic-ischemic brain injury. Pediatr Neurol. (2010) 43:236–40. 10.1016/j.pediatrneurol.2010.05.009 20837300

[25] Hofer N Zacharias E Müller W Resch B. An update on the use of C-reactive protein in early-onset neonatal sepsis: current insights and new tasks. Neonatology. (2012) 102:25–36. 10.1159/000336629 22507868

[26] Shephard EG Kelly SL Anderson R Fridkin M. Characterization of neutrophil-mediated degradation of human C-reactive protein and identification of the protease. Clin Exp Immunol. (1992) 87:509–13. 10.1111/j.1365-2249.1992.tb03028.x 1544236 PMC1554321

[27] Kunze R. C-reactive protein: from biomarker to trigger of cell death? Ther Apher Dial (2019) 23:494–6. 10.1111/1744-9987.12802 31788974

[28] Medzhitov R. Origin and physiological roles of inflammation. Nature. (2008) 454(7203):428–35. 10.1038/nature07201 18650913

[29] Hotamisligil GS. Inflammation and metabolic disorders. Nature. (2006) 444(7121):860–7. 10.1038/nature05485 17167474

[30] Barrett TD Hennan JK Marks RM Lucchesi BR. C-reactive-protein-associated increase in myocardial infarct size after ischemia/reperfusion. J Pharmacol Exp Ther. (2002) 303:1007–13. 10.1124/jpet.102.040600 12438521

[31] Chakkarapani E Davis J Thoresen M. Therapeutic hypothermia delays the C-reactive protein response and suppresses white blood cell and platelet count in infants with neonatal encephalopathy. Arch Dis Child Fetal Neonatal Ed. (2014) 99(6):F458–63. 10.1136/archdischild-2013-305763 24972990

[32] Banait T Wanjari A Danade V Banait S Jain J. Role of high-sensitivity C-reactive protein (hs-CRP) in non-communicable diseases: a review. Cureus. (2022) 14(10):e30225. 10.7759/cureus.30225 36381804 PMC9650935

[33] National Institute of Clinical Excellence (NICE). Therapeutic Hypothermia With Intracorporeal Temperature Monitoring for Hypoxic Perinatal Brain Injury. [Internet]. (2010). (Cited February 11, 2021).

[34] Ruhfus M Giannakis S Markus M Stein A Hoehn T Felderhoff-Mueser U Association of routinely measured proinflammatory biomarkers with abnormal MRI findings in asphyxiated neonates undergoing therapeutic hypothermia. Front Pediatr. (2021) 9:624652. 10.3389/fped.2021.624652 33855004 PMC8039151

[35] Antonelli M Kushner I. It's time to redefine inflammation. FASEB J. (2017) 31(5):1787–91. 10.1096/fj.201601326R 28179421

